# Investigation of the Antibacterial Properties and Mode of Action of Compounds From Urtica dioica L.

**DOI:** 10.7759/cureus.52083

**Published:** 2024-01-11

**Authors:** Juan Du, Jingyun Fu, Tao Chen

**Affiliations:** 1 Pharmaceutical Department, Yichang Yiling Hospital, Yichang, CHN; 2 Laboratory on Chinese Medicine Approved by State Administration of Traditional Chinese Medicine, China Three Gorges University, Yichang, CHN; 3 College of Medicine and Health Sciences, China Three Gorges University, Yichang, CHN; 4 College of Basic Medical Sciences, China Three Gorges University, Yichang, CHN

**Keywords:** methicillin resistant, chemical composition, staphylococcus aureus, antibacterial activity, urtica dioica

## Abstract

In recent years, the issue of antimicrobial resistance has gained significant global attention. The prevalence of methicillin-resistant *Staphylococcus aureus* (MRSA) infection rates worldwide has seen a rapid rise, increasing from 1%-5% in the mid-1980s to 60%-70% at present. This alarming increase in MRSA infection poses a serious threat to public health globally. Consequently, it is crucial to explore and identify effective drug candidates for combating MRSA. We researched the antibacterial properties of *Urtica dioica* L. Modern techniques such as systematic solvent extraction, macroporous resin chromatography, and silica gel column chromatography were utilized to isolate and detect components of MRSA. The most potent antibacterial active components were screened using fungal staining (K-B staining) and 2,3,5-triphenyltetrazolium chloride staining (TTC staining). Based on pharmacological activity guidance, we isolated a total of nine compounds from this plant. They were vanillic acid (compound A), quercetin-3-O-glucopyranoside (compound B), ursolic acid (compound C), vanillin (compound D), salicyl alcohol (compound E), kaempferol (compound F), quercetin (compound G), quercetin-3-O-galactoside (compound H), and isorhamnetin (compound I). Isolated compounds A, D, and E have better anti-MRSA activity. It inhibits bacterial division and growth during the logarithmic growth period and acts as a bacterial inhibitor. Inhibition may be mediated by the disruption of the bacterial cell structure, leading to leakage of contents including sugars, nucleic acids, and proteins. It may also be mediated by regulating phosphorus metabolism and disrupting the bacterial cell membrane potential to affect cellular metabolism.

## Introduction

Based on the most recent statistics provided by the World Health Organization, it is projected that over 700,000 individuals residing in Asian nations, including China, South Korea, and Malaysia, experience mortality annually due to the direct consequences of antibiotic resistance [1]. In Asian countries, antimicrobial resistance is reported to be increasing at an alarming rate [2]. The global rise and dissemination of antimicrobial resistance pose a significant challenge to public health, necessitating increased attention [3].

*Staphylococcus aureus* (SA) is representative of Gram-positive bacteria [4]. It can invade the body in various ways, causing various skin or organ infections [5,6]. Since the development and use of antibiotics such as penicillin and methicillin in the mid-20th century, the efficacy of treating *S. aureus* has been well established. Soon after, *S. aureus* showed rapid adaptation to these antimicrobial agents, resulting in the formation of methicillin-resistant *Staphylococcus aureus* (MRSA). Subsequently, the global prevalence of MRSA has increased dramatically, not only in healthcare settings but also in the community, posing a significant threat to global public health [7]. Many non-β-lactam antibiotics are recommended for the treatment of MRSA infections, including vancomycin, teicoplanin, linezolid, and apramycin [8,9]. Currently, vancomycin is widely acknowledged as the most efficacious pharmaceutical agent for the treatment of MRSA infection. Nevertheless, the therapeutic use of this substance is impeded by significant adverse effects such as ototoxicity and phlebitis resulting from its low pH. Additionally, the administration of vancomycin is constrained to intravenous injection, hence imposing limitations on its extensive utilization. Regrettably, the overall efficacy of vancomycin therapy remains suboptimal. Multiple investigations have shown evidence of the existence of vancomycin-resistant MRSA strains in various nations. Let us contemplate a potentially graver situation. The widespread development of vancomycin resistance in MRSA will pose significant challenges in the treatment of infected individuals, hence resulting in substantial adverse impacts on worldwide public health [10]. Therefore, research into effective and resistant anti-MRSA drug candidates is essential, and there is an urgent need to develop new antimicrobial agents for MRSA.

The perennial plant being examined in this study is a member of the Urticaceae family and is frequently employed as a traditional remedy in China. *Urtica dioica* L. possesses a diverse array of bioactive compounds, including phenols, lignans, flavonoids, coumarins, polysaccharides, volatile oil, sterols, and organic acids. These constituents have been found to display various pharmacological activities, such as antirheumatic, anti-inflammatory, analgesic, hypoglycemic, antibacterial, and antiviral effects [11]. In recent years, heterologous nettle has also been found to have anti-MRSA activity. Salehzadeh et al. studied the antibacterial activity of 16 strains of MRSA isolated from skin and wound infections, and the results showed that the methanol extract of nettle had significant antibacterial activity against MRSA [12]. The present findings indicate that urtica, sometimes referred to as nettle, exhibits promising properties as an antibacterial agent, hence warranting further investigation and advancement in scientific study. Presently, the existing body of research on specific constituents of urtica is minimal, therefore warranting more investigation into the antibacterial attributes of various *Urtica* species. Such exploration has the potential for significant contributions to the field. This study focuses on investigating the bacteriostatic activity of various strains of nettle through the utilization of several modern chemical separation and purification methods. These methods include thin layer chromatography (TLC), positive and negative phase silica gel column chromatography, gel column chromatography, thin layer preparation method, and semiliquid preparation. Additionally, the chemical composition of the antimicrobial active site is analyzed using nuclear magnetic resonance (NMR). The aim of this research is to identify a natural antibacterial drug that exhibits minimal toxicity and side effects while possessing a unique antibacterial mechanism. Furthermore, the findings of this study will provide theoretical support for future research and development in this field.

## Materials and methods

Experimental strains and medicinal materials

*Staphylococcus aureus* (resource code: BNCC186053, another serial number: ATCC25923) and methicillin-resistant *Staphylococcus aureus* (resource code: BNCC337371, another serial number: ATCC43300) were purchased from Beijing Beina Chuanglian Biotechnology Research Institute. The medicinal materials used in the experiment were purchased from Hubei and other places and were identified as *Urtica dioica* of the Urticaceae family.

PCR

The genomic DNA of activated bacteria was extracted, and specific genes of *Staphylococcus aureus* (16S rDNA, nuc gene, nucA gene) and methicillin-resistant *Staphylococcus aureus* (mecA gene) were selected for PCR detection. The design of primers was reported in two related literature, as shown in Table 1. After agarose gel electrophoresis and imaging with Bio-Rad gel scanning imaging system, the experimental results were observed and preserved.

**Table 1:** Primer sequences of 16S rDNA gene, nuc gene, nucA gene, and mecA gene of Staphylococcus aureus

Primer name	Primer sequence (5’-3’)	Fragment length (bp)
nucA F	CGC TTG CTA TGA TTG TGG TAG CC	239
nucA R	TTC GGT TTC ACC GTT TCT GGC G	
nuc F	TCG TCA AGG CTT GGC TAA AGT TGC	126
nuc R	TCA GCG TTG TCT TCG CTC CAA A	
mecA F	AAA ATC GAT GGT AAA GGT TGG C	533
mecA R	AGT TCT GCA GTA CCG GAT TTG C	
16S rDNA F	GTA GCG GTG AAA TGC GTA GAT A	160
16S rDNA R	GAA ACC CTC CAA CAC TTA GCA C	

K-B method

The drug sample (20 mg) is dissolved in 10 mL 5% dimethyl sulfoxide (DMSO) aqueous solution by ultrasonic assistance. If the water solubility is not good, the sample can be dissolved in DMSO first and then diluted. Several round blank filter papers with a diameter of 6 mm were prepared and packed into test tubes with plugs. They were autoclaved and sterilized at 121°C for 20 minutes. After drying, they were ready for use. A sterile liquid transfer gun was used to absorb 30 μL of drug solution under aseptic conditions, hit it on the blank filter paper prepared in advance, dry it, and set it aside. The liquid transfer gun was used to absorb 200 μL of bacterial solution with a concentration of 0.5 McDonnell unit in the ultraclean worktable and spread it in the prepared Mueller-Hinton broth (MHB) medium so that the bacterial liquid was evenly covered with the culture medium. The filter paper containing the drug to be tested was uniformly pasted on the surface of the culture plate (each piece is about 3 cm) and cultured in a constant temperature incubator at 37°C for 24 hours. It was observed that the effective drug could appear in a circle of non-growing bacteria in the area around the paper called the bacteriostatic zone. The larger the bacteriostatic circle, the stronger the bacteriostatic effect of the drug. 5% DMSO was used as the control group, and parallel experiments were conducted three times.

TTC staining

The bacterial suspension with good growth in the logarithmic phase was diluted with a Mueller-Hinton agar (MHA) medium. After adjusting the concentration of bacterial solution to about 106 colony-forming unit (cfu)/mL, 100 μL of bacterial suspension and drugs of different concentrations were added to the bacterial pore plate, and the final concentration was adjusted to 0.25, 0.5, 0.75, 1.75, and 2 mg/mL. Then, it was cultured in a constant temperature incubator at 37°C for 24 hours. After culture, 20 μL of 2,3,5-triphenyltetrazolium chloride was added to each experimental hole and mixed well. Then, it was placed in a constant temperature incubator to avoid light and cultured at 37°C for four hours, and the color changes in the experimental holes were observed. At the same time, with 5% DMSO as the control group, the maximum concentration of DMSO, which did not affect the growth of bacterial cells, was 5%, and parallel experiments were carried out three times.

Separation of compounds

After washing the sediment, the whole nettle herb was crushed into powder by a traditional Chinese medicine grinder and dried and preserved. First of all, 200 g of plant medicinal materials were extracted with water, methanol, and ethanol in the Soxhlet extraction device, two hours each time, three times. The extract was filtered twice with filter paper to remove the residual material. The extract was concentrated in a rotary evaporator, freeze-dried in a freeze dryer at -80°C, and stored in a beaker at -20°C until use. The extracts were screened for antibacterial activity in vitro, and the components with strong antibacterial activity were obtained. After separation and elution with D101 macroporous resin, different parts of the water layer, 30% ethanol layer, 60% ethanol layer, and 90% ethanol layer, were obtained. After screening the antibacterial activity in vitro, the components with strong antibacterial activity were obtained and separated, and eluted again by silica gel column chromatography. In the process of elution, a small amount of eluate was collected many times, and each eluent passed the TLC qualitative inspection, and the eluent with the same composition was merged and concentrated for standby. A relatively pure substance can usually be obtained by concentration, recrystallization, etc. If TLC examination found that there are still a variety of components, not completely eluted, the silica gel column can be reapplied to separate compounds.

Turbidimetry

After the bacteria were cultured in the logarithmic growth phase, the bacteria were transferred to the MHA medium containing drugs according to the ratio of 1:100 and then cultured in a constant temperature shaker at 37°C and 120 rpm. Samples were taken every three hours after culture, 200 μL each time. The growth curve of bacteria affected by drugs for different times was measured by OD600, and the growth curve of bacteria affected by drugs for different times was described by GraphPad. 5% DMSO was used as the control group, and parallel experiments were conducted three times.

Determination of total sugar

The bacterial suspension in the logarithmic growth phase was mixed with the drug with a 5 mL concentration of MIC in the same volume, mixed evenly, and then cultured in a constant temperature shaker at 37°C and 120 rpm. Then, samples were taken every two hours, 1 mL each time, and centrifuged at 11,000 rpm for two minutes. Then, the supernatant was absorbed and diluted with phosphate-buffered saline (PBS) five times, 50 μL was added to the Eppendorf (EP) tube with a liquid transfer gun, 200 μL anthrone reagent was added to it, and it was placed in ice water for five minutes at 0°C. Then, it was boiled for 10 minutes at 100°C and placed at room temperature for 10 minutes to cool. Taking the concentration and absorbance value of the standard glucose solution as the standard, the standard curve was drawn, and the absorbance value of the sample under 620 nm was determined by an enzyme-labeling instrument. 5% DMSO was used as the control group, and parallel experiments were conducted three times.

Determination of nucleic acid and protein content

The bacterial suspension was centrifuged at 4000 rpm for 15 minutes. The bacteria were collected and rinsed with PBS three times and then resuscitated in PBS. The 100 μL heavy suspension was added with 450 μL of different concentrations of drugs and cultured in a constant temperature incubator at 37°C for three hours. Then, the bacterial suspension was centrifuged at 8000 rpm for three minutes. The supernatant (200 μL) was taken, and its absorbance at 260 nm was determined by an enzyme-labeling instrument. The content of nucleic acid in bacterial suspension was the absorbance value. In addition, the Coomassie brilliant blue method was used to determine the content of protein in the supernatant. 5% DMSO was used as the control group, and parallel experiments were conducted three times.

Determination of phosphorus metabolism

The activated bacterial liquid was transferred to an MHA medium at 1:60 and cultured in a constant temperature shaker at 120 rpm at 37°C for 12 hours. Then, it was centrifuged at 5000 rpm for 10 minutes, the supernatant was absorbed and discarded, the bacterial suspension was diluted with PBS, and the bacterial concentration was adjusted to about 1×10^6^ cfu/mL. The bacterial solution of 0.5 mL and glucose solution were accurately mixed with a liquid transfer gun, mixed evenly, and then added to the centrifuge tube. Then, 200 μL of phosphorus standard solution and drug with the concentration of MIC were added to each of them. Samples were taken every two hours, 0.1 mL each time, and treated with trichloroacetic acid-ferrous sulfate and ammonium molybdate, and then, the absorbance of the sample under 630 nm was determined by an enzyme-labeling instrument. 5% DMSO was used as the control group, and parallel experiments were conducted three times.

Rhodamine 123 staining

The bacterial liquid with good growth in the logarithmic phase was diluted with an MHA medium, and the concentration of the bacterial solution was adjusted to about 1×107 cfu/mL. After adding the drug with the concentration of MIC, it was cultured in the shaker at 220 rpm for three hours and then rinsed with PBS three times. The configured Rh123 solution was added to the bacterial solution, and the final concentration was adjusted to MIC and then cultured in a constant temperature incubator at 37°C for 30 minutes. After that, the bacterial liquid was rinsed with PBS three times and then resuspended in PBS. The sample was added to the quartz colorimetric dish, and fluorescence intensity was measured using a fluorescence spectrophotometer. The excitation wavelength is 480 nm, and the emission wavelength is 530 nm. 5% DMSO was used as the control group, and parallel experiments were conducted three times.

Statistical analysis

Data were represented as mean±standard error of the mean (SEM), and statistical analysis was performed using the Statistical Package for the Social Sciences (SPSS) 18.0 (IBM SPSS Statistics, Armonk, NY) and GraphPad Prism 5.0 (GraphPad Software, Boston, MA). Comparison of two groups in multiple groups was performed using one-way analysis of variance (ANOVA) with Tukey's post hoc analysis. P<0.05 was considered statistically significant.

## Results

Activation of bacterial liquid

**Figure 1:**
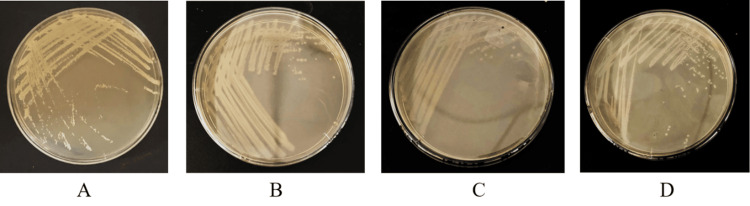
Plate plotting of bacteria A:​​​ SA, B: MRSA, C: ST5-MRSA, D: ST239-MRSA SA: *Staphylococcus aureus*, MRSA: methicillin-resistant *Staphylococcus aureus*

The result of the bacterial liquid plate marking is shown in Figure 1. The plate marking separation method of the bacterial liquid means that the mixed microorganisms or different cells in the same microbial population are diluted by the inoculation ring on the surface of the plate culture medium to obtain more independently distributed single cells, which grow and propagate into a single colony after culture. This single colony is usually regarded as a pure species of microorganisms to be isolated. In this subject, the strains were marked on the nutrient agar plate, and the obvious single colony could be seen. The results showed that the plate marking of the strain was successful.

Amplification of specific genes by PCR

**Figure 2:**
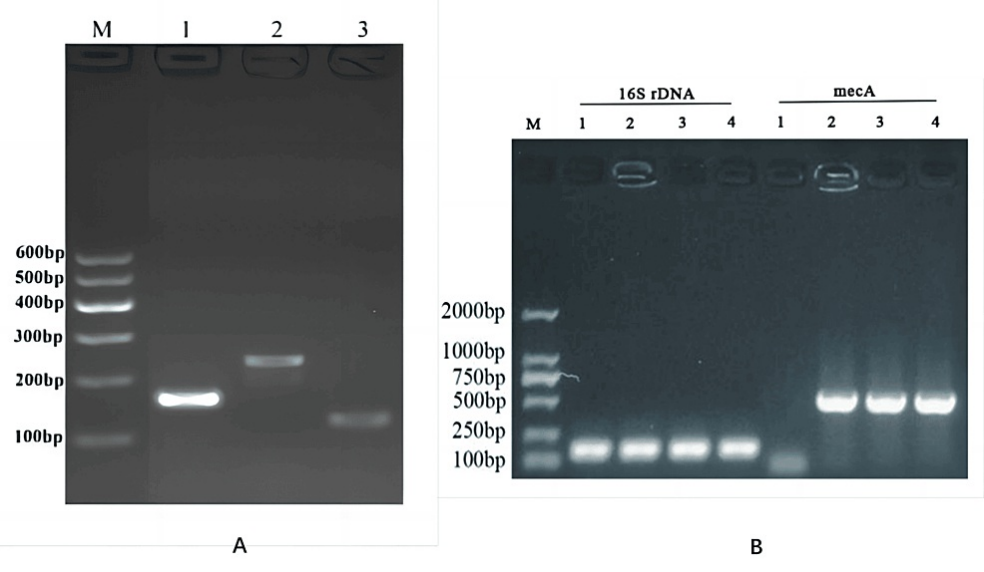
PCR-verified activation of SA, MRSA, ST5-MRSA, and ST239-MRSA strains A: Amplification of nuc, nucA, and 16S rDNA genes of *Staphylococcus aureus* by PCR. M-maker: 1: 16S rDNA, 2: nucA, 3: nuc B: Amplification of mecA and 16S rDNA genes of strains by PCR. M-maker: 1: SA, 2: ST5-MRSA, 3: ST239-MRSA, 4: MRSA PCR: polymerase chain reaction, SA: *Staphylococcus aureus*, MRSA: methicillin-resistant *Staphylococcus aureus*

16S rDNA gene can be used as a reference gene for the identification of SA. nuc and nucA genes are specific genes for the identification of SA. The length of the nuc fragment (126 bp) and nucA fragment (239 bp) amplified by SA is the same as that of the target fragment. mecA gene is a specific gene to identify MRSA. The length of the mecA fragment (533 bp) amplified by MRSA (MRSA, ST5-MRSA, ST239-MRSA) is consistent with that of the target fragment. The results showed that SA, MRSA, ST5-MRSA, and ST239-MRSA were activated successfully, as shown in Figure 2.

In vitro antibacterial activity of different solvent extracts of *Urtica dioica* against MRSA

As shown in Table 2 and Table 3, the water extract of *Urtica dioica* (WEU) could inhibit the growth of SA, ST5-MRSA, and ST239-MRSA, and the minimum inhibitory concentration (MIC) was 10, 15, and 15 mg/mL, respectively, but the WEU had no inhibitory effect on MRSA. The methanol extract of *Urtica dioica* (MEU) could inhibit the growth of SA, MRSA, ST5-MRSA, and ST239-MRSA, and the MIC was 5, 10, 10, and 15 mg/mL, respectively. The ethanol extract of *Urtica dioica* (EEU) could inhibit the growth of SA and ST5-MRSA, and the MIC was 10 and 15 mg/mL, respectively, but the EEU had no inhibitory effect on MRSA and ST239-MRSA. Compared with the MEU, the diameter of inhibition zone (DIZ) of SA, MRSA, and ST239-MRSA from the WEU and EEU decreased significantly (P<0.05 or P<0.01). The WEU showed the same effect on the DIZ of ST5-MRSA, and the difference was statistically significant (P<0.05 or P<0.01). The DIZ of EEU on ST5-MRSA decreased, but the difference was not statistically significant (P>0.05). Therefore, the MEU with stronger antibacterial activity was selected for further study.

**Table 2:** MIC of different solvent extracts from Urtica dioica to strains (mg.mL-1) MIC: minimum inhibitory concentration, SA: *Staphylococcus aureus*, MRSA: methicillin-resistant *Staphylococcus aureus*, WEU: water extract of *Urtica dioica*, MEU: methanol extract of *Urtica dioica*, EEU: ethanol extract of *Urtica dioica*

Sample	MIC of strains/mg.mL^-1^			
	SA	MRSA	ST5-MRSA	ST239-MRSA
WEU	10	＞20	15	15
MEU	5	10	10	15
EEU	10	＞20	15	＞20

**Table 3:** DIZ of different solvent extracts from Urtica dioica to strains (mm) "N" means no effect, compared with MEU. *P<0.05, **P<0.01 DIZ: diameter of inhibition zone, WEU: water extract of *Urtica dioica*, MEU: methanol extract of *Urtica dioica*, EEU: ethanol extract of *Urtica dioica*,SA: *Staphylococcus aureus*, MRSA: methicillin-resistant *Staphylococcus aureus*

Strains	WEU	MEU	EEU	Negative control	Positive control
SA	9.37±1.42^**^	14.10±0.90	11.87±0.90^*^	N	18.63±1.61
MRSA	9.97±1.85^**^	13.80±0.79	11.00±0.92^*^	N	19.33±0.57
ST5-MRSA	9.23±1.07^*^	12.57±0.59	10.67±1.95	N	18.53±1.10
ST239-MRSA	9.90±0.90^**^	13.33±1.46	11.23±0.40^*^	N	19.57±0.51

Separation of MEU by D101 macroporous adsorption resin the in vitro antibacterial activity of the eluted components against MRSA

As shown in Table 4 and Table 5, the water layer of MEU (WMEU) could inhibit the growth of SA and ST5-MRSA, and the MIC was 10 and 15 mg/mL, respectively, but it had no inhibitory effect on MRSA and ST239-MRSA. The 30% ethanol layer of MEU (30%-EMEU) could inhibit the growth of SA, MRSA, ST5-MRSA, and ST239-MRSA, and the MIC was 10, 15, 15, and 15 mg/mL, respectively. The 60% ethanol layer of MEU (60%-EMEU) could inhibit the growth of SA, MRSA, ST5-MRSA, and ST239-MRSA, and the MIC was 10, 10, 20, and 15 mg/mL, respectively. The 90% ethanol layer of MEU (90%-EMEU) could inhibit the growth of SA, and the MIC was 10 mg/mL, but it had no inhibitory effect on MRSA, ST5-MRSA, and ST239-MRSA.

**Table 4:** MIC of eluent components by D101 macroporous adsorption resin from Urtica dioica to strains (mg.mL-1) MIC: minimum inhibitory concentration, SA: *Staphylococcus aureus*, MRSA: methicillin-resistant *Staphylococcus aureus*, WMEU: water layer of the methanol extract of *Urtica dioica*, 30%-EMEU: 30% ethanol layer of the methanol extract of *Urtica dioica*, 60%-EMEU: 60% ethanol layer of the methanol extract of *Urtica dioica*, 90%-EMEU: 90% ethanol layer of the methanol extract of *Urtica dioica*

Sample	MIC of strains/mg.mL^-1^			
	SA	MRSA	ST5-MRSA	ST239-MRSA
WMEU	10	＞20	15	＞20
30%-EMEU	10	15	15	15
60%-EMEU	10	10	20	15
90%-EMEU	15	＞20	＞20	＞20

**Table 5:** DIZ of eluent components by D101 macroporous adsorption resin from Urtica dioica to strains (mm) "N" means no effect, compared with 30%-EMEU. #P<0.05 and ##P<0.01, compared with 60%-EMEU *P<0.05, **P<0.01 DIZ: diameter of inhibition zone, WMEU: water layer of the methanol extract of *Urtica dioica*, 30%-EMEU: 30% ethanol layer of the methanol extract of *Urtica dioica*, 60%-EMEU: 60% ethanol layer of the methanol extract of *Urtica dioica*, 90%-EMEU: 90% ethanol layer of the methanol extract of *Urtica dioica*, SA: *Staphylococcus aureus*, MRSA: methicillin-resistant *Staphylococcus aureus*

Strains	WMEU	30%-EMEU	60%-EMEU	90%-EMEU	Negative control	Positive control
SA	9.97±0.06^#*^	13.03±1.00	12.30±1.1.3	8.90±1.01^##**^	N	18.67±1.55
MRSA	9.23±0.32^##**^	12.13±0.91	12.67±1.15	9.53±1.36^#**^	N	19.27±0.49
ST5-MRSA	9.93±0.90^#^	12.47±1.65	11.83±1.46	8.67±1.15^##*^	N	18.37±0.81
ST239-MRSA	8.67±0.58^##**^	11.43±0.67	10.80±1.06	8.33±0.58^##**^	N	19.53±0.47

Compared with 30%-EMEU, the DIZ of SA, MRSA, ST5-MRSA, and ST239-MRSA decreased significantly in WMEU and 90%-EMEU (P<0.05 or P<0.01), the DIZ of SA, ST5-MRSA, and ST239-MRSA decreased in 60%-EMEU, but the DIZ of MRSA became larger, and the difference was not statistically significant (P>0.05). Compared with 60%-EMEU, the DIZ of SA, MRSA, and ST239-MRSA decreased significantly in WMEU and 90%-EMEU (P<0.05 or P<0.01), 90%-EMEU had the same effect on ST5-MRSA, and the difference was statistically significant (P<0.05 or P<0.01). The DIZ of WMEU to ST5-MRSA became smaller, but the difference was not statistically significant (P>0.05). The results showed that the anti-MRSA active sites were mainly concentrated in 30%-EMEU and 60%-EMEU, so these two components were selected for further study.

Structural analysis and identification of compounds

**Figure 3:**
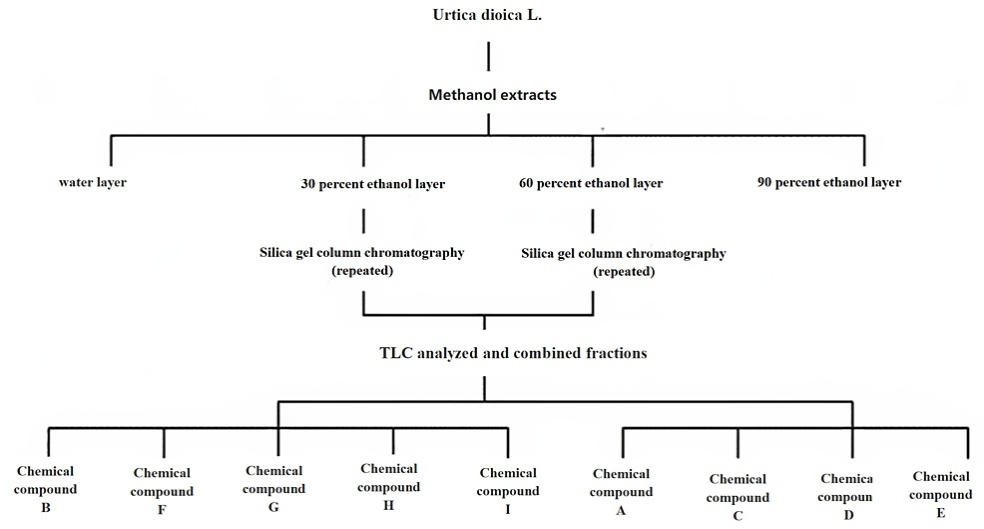
Isolation of anti-MRSA active site from Urtica dioica L. MRSA: methicillin-resistant *Staphylococcus aureus*

Figure 3 illustrates the isolation of nine monomer molecules from 30%-EMEU and 60%-EMEU using contemporary chemical separation techniques.

The carbon-13 nuclear magnetic resonance (^13^C-NMR) results of each monomer compound are analyzed as follows. Compound A was identified as vanillic acid [13]. It appeared as a white powder. Compound B was identified as quercetin-3-O-glucoside, which appeared as a yellow powder [14]. Compound C was identified as ursolic acid and appeared as a white powder [15]. Compound D was identified as vanillin, which appeared as white needle-like crystals [16]. Compound E was identified as salicyl alcohol in the form of white granules [17]. Compound F appeared as a yellow powder and was identified as kaempferol [18]. Compound G was identified as quercetin and appeared as a yellow powder [19]. Compound H was identified as quercetin-3-O-galactoside (hypericin) and appeared as a yellow powder [20]. Compound I was identified as isorhamnetin and appeared as a yellow powder [21]. The ^13^C-NMR data (400MHz, DMSO) of all the compounds are presented in Table 6.

**Table 6:** 13C-NMR data of compounds A, B, C, D, E, F, G, H, and I ^13^C-NMR: carbon-13 nuclear magnetic resonance

Chemical shift δ (ppm)	Assignment
Compound A (vanillic acid)	
55.97	OCH_3_
113.15	C-2
115.49	C-5
122.07	C-1
123.95	C-6
147.66	C-3
151.53	C-4
167.68	COOH
Compound B (quercetin-3-O-glucoside)	
61.43	C-6''
70.39	C-4''
74.55	C-2' '
76.95	C-3''
78.03	C-5''
93.95	C-8
99.11	C-6
101.3	C-1 ''
104.44	C-10
115.66	C-2'
116.66	C-6'
121.62	C-1'
122.06	C-5 '
133.77	C-3
145.26	C-3'
148.9	C-4'
156.63	C-2
156.77	C-9
161.69	C-5
164.54	C-7
177.9	C-4
Compound C (ursolic acid)	
15.7	C-26
16.56	C-25
17.39	C-24
17.49	C-6
18.47	C-29
21.55	C-16
23.32	C-2
23.74	C-11
24.27	C-30
27.46	C-27
28.01	C-21
28.73	C-20
30.65	C-19
33.17	C-15
36.78	C-7
37	C-22
38.69	C-10
38.85	C-1
38.9	C-8
38.97	C-4
42.11	C-14
47.29	C-17
47.48	C-9
52.84	C-5
55.24	C-18
77.27	C-3
125.04	C-12
138.65	C-13
178.72	C-28
Compound D (vanillin)	
56.04	OCH_3_
111.14	C-2
115.84	C-5
126.52	C-6
129.17	C-1
148.61	C-3
153.47	C-4
191.44	CHO
Compound E (salicyl alcohol)	
58.69	C-7
114.95	C-3
119.1	C-5
127.75	C-4
128.97	C-6
154.57	C-2
Compound F (kaempferol)	
93.94	C-8
98.67	C-6
103.5	C-10
115.9	C-3'
115.9	C-5'
122.13	C-1 '
129.96	C-2'
129.96	C-6'
136.12	C-3
147.26	C-2
156.63	C-9
159.64	C-4'
161.17	C-5
164.35	C-7
176.35	C-4
Compound G (quercetin)	
93.79	C-8
98.62	C-6
103.46	C-10
115.51	C-2'
116.05	C-5'
120.42	C-1'
122.4	C-6'
136.18	C-3
145.5	C-3'
147.24	C-2
148.15	C-4'
156.58	C-5
161.17	C-9
164.32	C-7
176.28	C-4
Compound H (quercetin-3-O-galactoside (hypericin))	
60.6	C-6''
68.38	C-4''
71.66	C-2''
73.64	C-3''
76.3	C-5''
93.95	C-8
99.12	C-6
102.23	C-1 ''
104.38	C-10
115.63	C-2''
116.39	C-5''
121.55	C-1'
122.46	C-6 '
133.93	C-3
145.28	C-3'
148.91	C-4'
156.68	C-2
156.75	C-9
161.68	C-5
164.56	C-7
177.94	C-4
Compound I (isorhamnetin)	
56.23	C-1''
94.95	C-8
98.66	C-6
103.49	C-10
112.17	C-2'
116	C-5'
122.18	C-6'
122.42	C-1'
136.28	C-3
147.08	C-2
147.81	C-3'
149.26	C 4'
156.61	C-9
161.14	C-5
164.36	C-7
176.33	C-4

**Figure 4:**
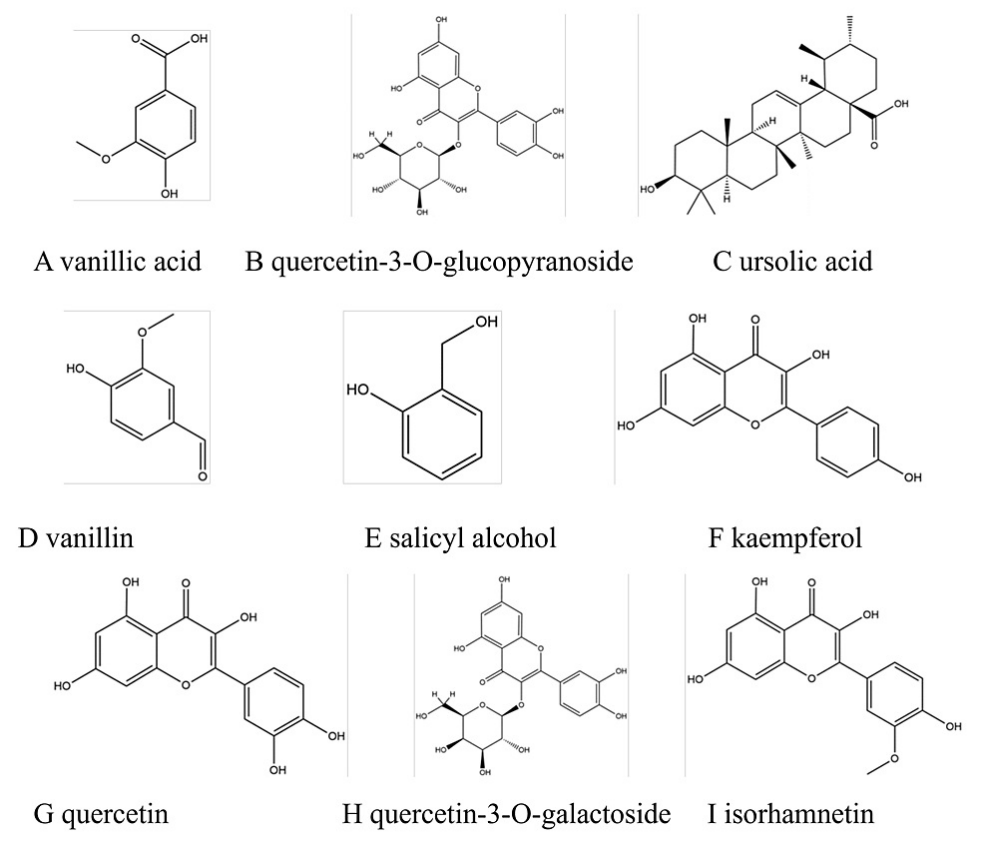
Chemical structure diagram of compounds A: vanillic acid, B: quercetin-3-O-glucopyranoside, C: ursolic acid, D: vanillin, E: salicyl alcohol, F: kaempferol, G: quercetin, H: quercetin-3-O-galactoside, I: isorhamnetin

Figure 4 displays the chemical structure of the nine monomer compounds in their final forms.

In vitro antibacterial activity of compounds against MRSA

The findings shown in Table 7 demonstrate that compound A-I exhibits varying inhibitory effects on distinct strains of bacteria, including SA, MRSA, ST5-MRSA, and ST239-MRSA. The minimum inhibitory concentration (MIC) of compound A-I against SA is seen to be 1, 2, 0.5, 1, 1.75, 1.5, 1, and >2 mg/mL, respectively. Similarly, the MIC of compound A-I against MRSA is found to be 1, 2, 0.75, 1.5, 1.75, >2, >1, and >2 mg/mL, respectively. The minimum inhibitory concentration (MIC) values for ST5-MRSA were 1, >2, 0.5, 1, 1.5, 0.25, >2, 1.5, and >2 mg/mL, whereas the MIC values for ST239-MRSA were 1, >2, 0.75, 1, 1.5, 0.25, >2, >2, and 2 mg/mL, respectively.

**Table 7:** MIC of compounds to strains (mg.mL-1) MIC: minimum inhibitory concentration, SA: *Staphylococcus aureus*, MRSA: methicillin-resistant *Staphylococcus aureus*

Sample	MIC of strains/mg.mL^-1^			
	SA	MRSA	ST5-MRSA	ST239-MRSA
Compound A	1	1	1	1
Compound B	2	＞2	＞2	＞2
Compound C	0.5	0.75	0.5	0.75
Compound D	1	1.5	1	1
Compound E	1.75	1.75	1.5	1.5
Compound F	1.5	＞2	0.25	0.25
Compound G	1.75	＞2	＞2	＞2
Compound H	1	1	1.5	＞2
Compound I	＞2	＞2	＞2	2

**Figure 5:**
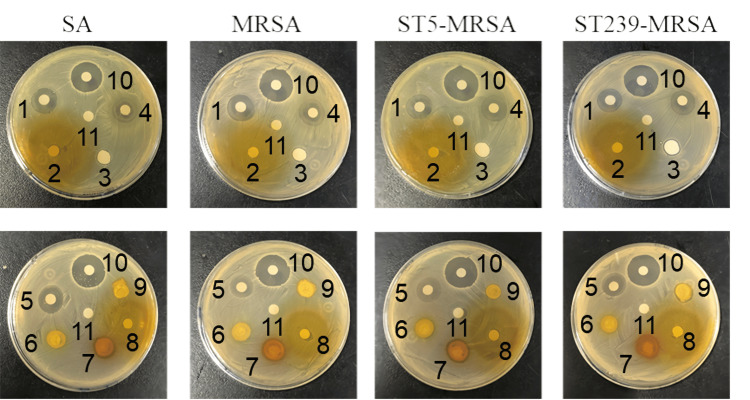
DIZ of compounds to strains 1: compound A, 2: compound B, 3: compound C, 4: compound D, 5: compound E, 6: compound F, 7: compound G, 8: compound H, 9: compound I, 10: positive control, 11: negative control DIZ: diameter of inhibition zone, SA: *Staphylococcus aureus*, MRSA: methicillin-resistant *Staphylococcus aureus*

The inhibitory effects of compounds A-I on SA, ST5-MRSA, and ST239-MRSA were observed and documented in Table 8 and Figure 5, demonstrating varied degrees of inhibition. In comparison to compound A, compounds B-I exhibited a notable drop in the DIZ (P<0.05 or P<0.01) when tested against SA. The diameter of inhibition zone (DIZ) of compound D against methicillin-resistant *Staphylococcus aureus* (MRSA) and ST5-MRSA exhibited a reduction; however, this drop did not reach statistical significance (P>0.05). The DIZ of other drugs against MRSA and ST5-MRSA exhibited a substantial reduction, with statistical significance seen at a significance level of P<0.05 or P<0.01. The reduction in the DIZ of compound D and compound E against ST239-MRSA was observed; however, the observed difference did not reach statistical significance. The DIZ of other drugs exhibited a substantial reduction in the presence of ST239-MRSA, as shown by statistical significance at the P<0.05 or P<0.01 level. Hence, compounds A, D, and E exhibiting enhanced antibacterial efficacy were chosen for subsequent investigation.

**Table 8:** DIZ of compounds to strains (mm) "N" means no effect, compared with compound 1. *P<0.05, **P<0.01 DIZ: diameter of inhibition zone, SA:* Staphylococcus aureus*, MRSA: methicillin-resistant *Staphylococcus aureus*

Strains	SA	MRSA	ST5-MRSA	ST239-MRSA
Compound A	14.90±0.17	15.63±0.38	15.53±0.47	14.67±0.59
Compound B	6.67±0.59^**^	N	N	N
Compound C	7.77±0.49^**^	7.63±0.60^**^	9.57±0.32^**^	10.93±0.25^**^
Compound D	12.07±0.90^**^	14.90±0.26	15.67±0.06	14.63±0.12
Compound E	14.33±0.42	13.33±0.42^**^	14.57±0.40^*^	14.57±0.51
Compound F	9.67±0.42^**^	10.33±0.42^**^	11.40±0.53^**^	10.60±0.53^**^
Compound G	10.70±0.36^**^	10.93±0.90^**^	13.27±0.64^**^	12.33±1.15^**^
Compound H	5.60±0.53^**^	N	N	N
Compound I	7.40±0.40^**^	9.30±0.36^**^	10.00±0.92^**^	11.00±0.20^**^
Negative control	N	N	N	N
Positive control	19.30±0.61	19.33±0.58	18.97±1.00	18.97±1.05

Inhibitory effect of compounds on the bacterial growth curve

**Figure 6:**
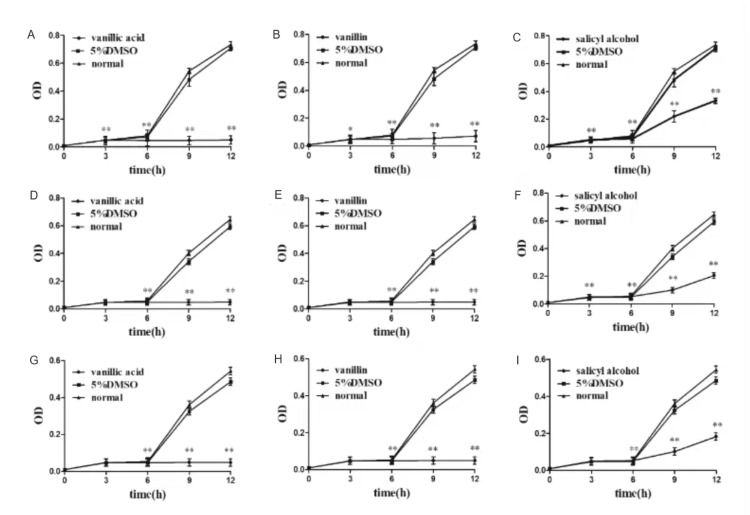
Inhibitory effect of compounds on bacterial growth curve A-C: vanillic acid, vanillin, and salicyl alcohol on MRSA, D-F: vanillic acid, vanillin, and salicyl alcohol on ST5-MRSA, G-I: vanillic acid, vanillin, and salicyl alcohol on ST239-MRSA, compared with the normal group *P<0.05, **P<0.01 MRSA: methicillin-resistant *Staphylococcus aureus*, DMSO: dimethyl sulfoxide

The results are shown in Figure 6. After a short lag period, the bacteria without drugs can quickly enter the exponential phase of bacterial growth. However, in the experimental group with monomer compounds, the exponential growth period of bacteria was significantly inhibited (P<0.05 or P<0.01), and the growth of bacteria was always at a low level. The results showed that vanillic acid, vanillin, and salicyl alcohol might exert a bacteriostatic effect by inhibiting cell division and growth in the logarithmic growth phase.

Effect of compounds on the concentration of total sugar in the bacterial solution

**Figure 7:**
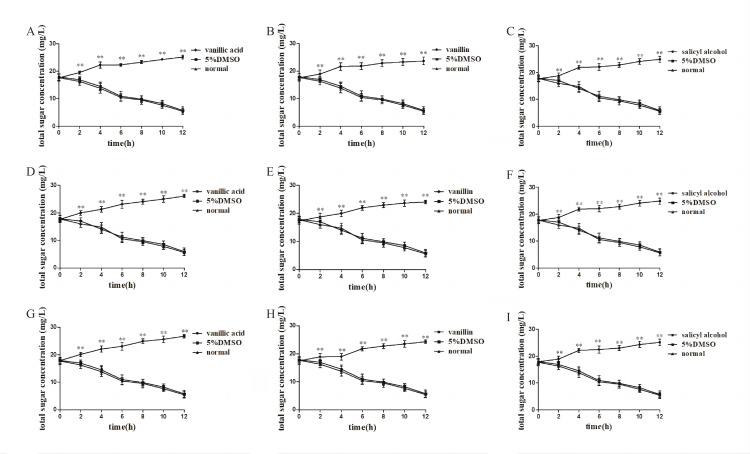
Effect of compounds on the concentration of total sugar in bacterial solution A-C: vanillic acid, vanillin, and salicyl alcohol on MRSA, D-F: vanillic acid, vanillin, and salicyl alcohol on ST5-MRSA, G-I: vanillic acid, vanillin, and salicyl alcohol on ST239-MRSA, compared with the normal group *P<0.05, **P<0.01 MRSA: methicillin-resistant *Staphylococcus aureus*, DMSO: dimethyl sulfoxide

The result is shown in Figure 7. The normal group showed a decrease in the concentration of total sugar in the culture medium, which was due to the need for normal bacteria to absorb and utilize the sugars in the culture medium during proliferation. However, when the bacteria acted with the drug, the concentration of total sugar in the culture medium gradually increased with the extension of the action time, and the difference was statistically significant (P<0.05 or P<0.01). The results showed that the sugars in the bacterial cells gradually leaked out of the cells, and the cell membrane structure of the bacteria may have been destroyed. Therefore, vanillic acid, vanillin, and salicyl alcohol may cause cytoplasmic outflow by destroying the cell structure of bacteria, thus inhibiting the reproduction and growth of bacteria.

Effect of compounds on the content of nucleic acid and protein of bacteria

As shown in Table 9 and Table 10, when bacteria were treated with drugs, compared with the normal group, the absorbance of bacterial suspension under 260 nm increased, and the difference was statistically significant (P<0.05 or P<0.01), and the content of nucleic acid increased. Compared with the normal group, the protein content in bacterial suspension was the same after the interaction of bacteria with drugs, and the difference was statistically significant (P<0.05 or P<0.01). Thus, it can be seen that vanillic acid, vanillin, and salicyl alcohol may destroy the integrity of bacterial cell structure, resulting in the outflow of nucleic acid and protein into the culture medium and inhibiting the growth and reproduction of bacteria.

**Table 9:** Effects of compounds on the release of bacterial nucleic acid compared with normal *P<0.05, **P<0.01 MRSA: methicillin-resistant *Staphylococcus aureus*

A_260nm_	Vanillic acid	Vanillin	Salicyl alcohol	Normal	5% DMSO
MRSA	0.39±0.05^**^	0.35±0.06^**^	0.33±0.04^**^	0.02±0.01	0.01±0.00
ST5-MRSA	0.40±0.06^**^	0.33±0.06^**^	0.30±0.07^**^	0.01±0.01	0.01±0.01
ST239-MRSA	0.48±0.13^**^	0.36±0.06^**^	0.33±0.05^**^	0.02±0.01	0.01±0.00

**Table 10:** Effects of compounds on the release of bacterial protein compared with normal *P<0.05, **P<0.01 MRSA: methicillin-resistant *Staphylococcus aureus*

Protein (µg/mL)	Vanillic acid	Vanillin	Salicyl alcohol	Normal	5% DMSO
MRSA	63.69±7.90^**^	58.89±5.84^**^	48.44±7.48^**^	10.70±1.40	9.42±1.53
ST5-MRSA	61.80±6.65^**^	55.30±6.50^**^	47.89±3.73^**^	10.40±0.89	10.34±1.14
ST239-MRSA	58.63±8.21^**^	54.41±5.01^**^	46.20±4.16^**^	9.60±1.30	8.83±1.00

Effect of compounds on phosphorus metabolism of bacteria

**Figure 8:**
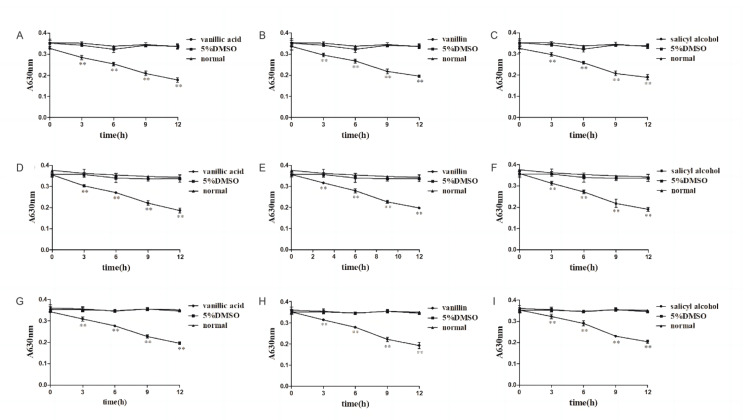
Effect of compounds on phosphorus metabolism of bacteria A-C: vanillic acid, vanillin, and salicyl alcohol on MRSA, D-F: vanillic acid, vanillin, and salicyl alcohol on ST5-MRSA, G-I: vanillic acid, vanillin, and salicyl alcohol on ST239-MRSA, compared with the normal group *P<0.05, **P<0.01 MRSA: methicillin-resistant *Staphylococcus aureus*, DMSO: dimethyl sulfoxide

As shown in Figure 8, compared with the normal group, when the bacteria were treated with drugs at different times, the phosphorus consumption of the bacteria decreased, and the difference was statistically significant (P<0.05 or P<0.01), which indicated that the phosphorus metabolism of the bacteria was out of order. Thus, it can be seen that vanillic acid, vanillin, and salicyl alcohol may exert a bacteriostatic effect by affecting cell metabolism.

Effect of compounds on cell membrane potential

**Figure 9:**
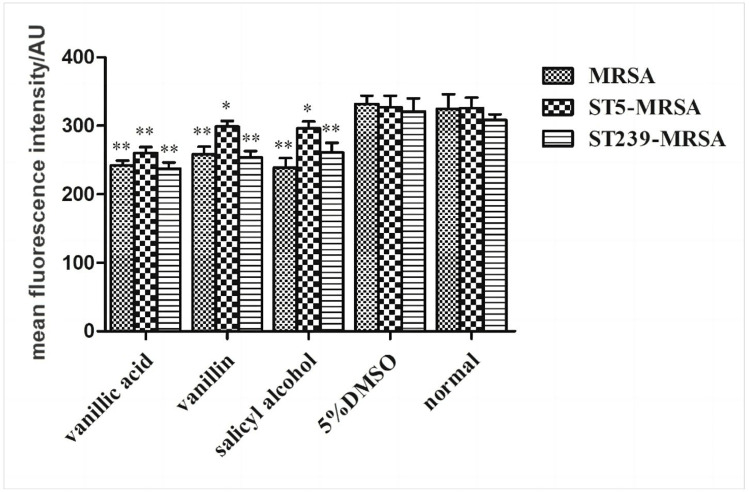
Effect of compounds on cell membrane potential compared with the normal group *P<0.05, **P<0.01 MRSA: methicillin-resistant *Staphylococcus aureus*, DMSO: dimethyl sulfoxide

Membrane potential is the ion potential of different concentrations formed on both sides of the normal cell membrane. Under normal circumstances, Rh123 will enter the bacterial cell through the cell membrane and selectively stain the mitochondria in the living cell. However, when the drug reacted with bacteria, as shown in Figure 9, compared with the normal group, the release of Rh123 and the average fluorescence intensity in the bacterial solution decreased, and the difference was statistically significant (P<0.05 or P<0.01), which indicated that the drug caused the depolarization of the cell membrane potential and destroyed the membrane potential of the bacterial cell. Thus, it can be seen that vanillic acid, vanillin, and salicyl alcohol may play a bacteriostatic effect by reducing the cell membrane potential and affecting the metabolic activity of bacterial cells.

## Discussion

Recently, infection has been an important contributor to morbidity and mortality in hospital settings and in the community [22]. The misuse of antibiotics to treat infections leads to the development of antibiotic resistance in pathogens, and a common phenomenon in which antibiotic therapy fails is SA infection. SA is a Gram-positive, partially anaerobic bacterium and is the predominant staphylococcal species in clinical practice [23]. The bacterium was first isolated in 1880 by the Scottish surgeon Alexander Augston, primarily from specimens of infectious abscesses [24]. It is estimated that about 30% of humans are asymptomatic carriers of this pathogen [25]. Most MRSA strains contain the mecA gene located on the staphylococcal cassette chromosome mec (SCCmec), which encodes penicillin-binding protein 2a (PBP2a) [26,27]. The presence of MRSA-specific PBP2a replaces inactivated penicillin-binding proteins and promotes further synthesis of the *S. aureus* cell wall and eventual resistance. To determine the presence of methicillin-resistant *S. aureus* and its specific resistance gene mecA, we performed PCR experiments to confirm that the length of the amplified mecA fragment (533 bp) was consistent with the length of the target fragment.

Understanding the clonal typing of MRSA, differentiation methods, and the genetic information and relationships between different strains are essential in the study of MRSA. Numerous studies have demonstrated that the epidemic strain types of MRSA in China are ST239 and ST5. These typing methods play a crucial role in tracking and managing the spread of MRSA infections [28,29]. Based on the available information, the susceptible strains selected for our investigation are ST5-MRSA and ST239-MRSA [30].

The research and progress of antimicrobial bioactive compounds in the herbal class is of great importance in addressing the problem of drug resistance, and several research programs have provided evidence that herbs have an anti-MRSA effect, but the extent of their inhibitory effect may vary, and several national and international studies have demonstrated the inhibitory capacity of nettle, as well as its specific inhibitory effect on MRSA [31,32]. In this investigation, 3 kg of nettles were used in the separation process, and the extraction rate reached 10%. In the subsequent separation process, the efficacy tracking method was used. Through the use of MIC and DIZ tests, the research has achieved remarkable results. The results showed that the antibacterial effect of the three phenolic compounds on MRSA was significantly enhanced, which was consistent with the results of many studies at home and abroad.

The medicine derived from *Urtica dioica* heterophylla was investigated for its inhibitory impact on bacteria using minimum inhibitory concentration (MIC) and disc diffusion (DIZ) assays. The results demonstrated its potential as a candidate for the development of an anti-MRSA infection treatment. The present study aimed to examine the impact of pharmaceutical substances on the proliferation of bacteria by the utilization of turbidimetry. The growth curve of bacteria serves as a valuable tool for elucidating the dynamic progression of bacteria in a cultured environment. This curve provides insights into several aspects of bacterial behavior, encompassing growth, reproduction, senescence, and apoptosis, to a certain degree. The growth curve exhibited by each variety of bacteria is distinct from that observed in other microorganisms, thereby rendering it unique. The findings indicated that the introduction of the medicine resulted in a reduced level of bacterial growth in comparison to the control group, thereby demonstrating the drug's inhibitory impact on bacterial growth.

A review of the literature suggests that the antibacterial activity of many Chinese herbal extracts may be due to their ability to disrupt the cellular structure of bacteria [33]. To investigate the impact of medications on cell structure, the amounts of sugars, nucleic acids, and proteins in bacterial suspensions were assessed subsequent to drug-bacteria interactions. The preservation of cellular homeostasis is intricately associated with the safeguarding of the bacterial cell membrane. Simultaneously, the cell membrane assumes a crucial function in facilitating the interchange of materials and energy between cells and the external environment [34,35]. The destruction of the cell membrane results in the release of intracellular chemicals into the surrounding culture media, leading to a significant impairment in cellular function. Polysaccharides play a crucial role in the bacterial growth and reproduction process, since bacteria often acquire sugars from the surrounding environment, such as a culture medium, and utilize them as nourishment for their growth. As seen in Figure 7, the collective concentration of polysaccharides in the control group exhibited a declining pattern, which aligns with the aforementioned statement. Nevertheless, it was observed that the experimental group exhibited an elevation in the content of polysaccharides within the culture media. Additionally, there was a progressive release of intracellular polysaccharides from the cells, suggesting that the administered medication had the capability to disrupt the integrity of the cell membrane and therefore induce leakage of the cytoplasm. Macromolecular compounds, such as nucleic acids and proteins, are fundamental constituents found in all known species and exhibit extensive cellular distribution. The activities of all known life forms are intrinsically linked to proteins, serving as their fundamental building blocks. Proteins play a crucial role in maintaining cellular osmotic pressure equilibrium and supplying energy for many living processes. Due to the fact that nucleic acid and protein constitute biological macromolecules, the process of bacterial penetration through the cell membrane and cell wall during regular growth is rendered challenging. Detecting nucleic acid and protein in a bacterial solution might be challenging until the integrity of the bacteria's cell structure is compromised.

Phosphorus is a crucial micronutrient inside the human body. In order to examine the impact of the medications on the comprehensive metabolic function and growth status of the bacterial cells, we conducted measurements of the phosphorus content pertaining to the metabolic activity subsequent to the introduction of the pharmaceuticals. The findings indicated that, in comparison to the control group, the experimental group exhibited a gradual decline in phosphorus consumption by bacterial cells as the duration of exposure increased. This suggests that the drug may exhibit antibacterial properties by influencing the phosphate metabolism of bacterial cells, consequently impacting cellular metabolism. In order to establish a stronger correlation between medications and cellular metabolism, we employed the utilization of the fluorescent dye Rh123 to examine alterations in membrane potential subsequent to drug delivery. Rh123 is a lipophilic cationic fluorescent probe that is widely employed for the purpose of labeling cells. The relationship between intracellular ATP levels and the fluorescence intensity of Rh123 is well-established. Rh123 is a specific dye that can effectively stain the mitochondria of viable cells. Consequently, alterations in membrane potential may be directly linked to changes in the fluorescence intensity of Rh123. As seen in Figure 9, within the control group, the bacterial cells experience the penetration of Rh123 via the cell membrane. Nevertheless, upon exposure to the medication, these bacteria experience depolarization of the cell membrane potential, which in turn disrupts the membrane potential of the bacterial cell, resulting in the subsequent release of Rh123. Consequently, this process leads to a decrease in the overall fluorescence intensity. The aforementioned data indicate a potential correlation between the bacteriostatic action of medications and their influence on the metabolism of bacterial cells.

## Conclusions

In summary, our study findings indicate that *Urtica dioica* L. exhibits promising potential in the context of combating MRSA infections. Based on the effectiveness monitoring approach, a total of nine monomeric compounds have been successfully identified, each exhibiting varying degrees of bacteriostatic activity. This medical resource is anticipated to serve as a potential medication candidate for combating MRSA infections.
